# Annotations

**Published:** 1889-11-30

**Authors:** 


### Annotations.

**A Privilege of the Rich.** — The gift of Sir Edward Guinness of a quarter of a million sterling for the erection and management of sanitary and comfortable workmen's dwellings, is a hopeful sign of the times. We belong to the class of economists who recognise the value of capital and the merits of those who accumulate and make use of it. But we also see very clearly that labour is the fundamental condition, not only of all human improvement, but even of the continuance of life itself. A quarrel between labour and capital is the fratricidal strife of those who are bound by every consideration of life, of happiness, and of progress to agree and be at one. Yet such a quarrel is by no means rare. On the other hand, there have always been a few mediators between capital and labour; and the number and influence of these wisest of all economists is constantly on the increase. This gift of a quarter of a million by Sir Edward Guinness will do more to bring labour and capital together than cartloads of musty treatises. It will do this because it will help to break down at once and effectually the solid barrier of distrust and suspicion which labour is always inclined to feel against capital. Any working-man of the least sense in the world must say to himself, when he considers Sir Edward's gift, that whatever else the donor may lack, he at least is possessed of a deep and strong sympathy with one of the most serious drawbacks of a working-man's life. Whatever other rich men may feel and think, it is plain that this rich man feels that a workman who has to live in a room too small for health, in a house too limited for decency and self-respect, in a soot-begrimed, dirt-encrusted, polluted, and miserable sty of a dwelling, is not very likely to be either happy, or virtuous, or pleased with his fellows. One of the greatest possible benefits, political or social, which could be conferred upon this country would be the erection in wholesome and pleasant neighbourhoods of a sufficient number of sanitary and comfortable workmen's dwellings. It would work more practical good than most of the measures which parliamentarians struggle and fight over. Among the most inestimable of the privileges of the rich is this, that, without Act of Parliament or voluntary committee, they can promptly and immediately set in operation the mediating forces of sympathy and practical good work. The destination of Sir Edward's splendid gift is a workmen's dwellings scheme, but that is to be so administered as to be almost or entirely self-supporting. What we understand is that the quarter of a million is to supply the initial capital for a great movement without cost. Nothing could be better. By all means let workmen have good and healthy houses; but let them pay reasonably for them. Private enterprise cannot, or at least does not, supply them with such houses. Rich men like Sir Edward Guinness may render incalculable service to their country by coming in at the right time, and effecting by generous gifts what private enterprise so conspicuously fails to do. It is much to be desired that many other wealthy men will give earnest thought to the subject, and consider if it would not be well for them to follow Sir Edward's most noble and generous example.

**A Debtor to his Profession.** — "I hold every man a debtor to his profession," says Bacon, "from the which as men, of course, do seek to receive countenance and profit, so ought they of duty to endeavour themselves, by way of amends, to be a help and an ornament thereunto." From the brightest luminary of the purest heavens down to the most miserable scamp of the professional slums, every professional man will echo this sentiment, as a sentiment. When the point of practice is reached there is a most terrible falling off. Medical men seem to set immense store by the professional spirit and the observance of professional rules; but it would appear, as a matter of fact, that there is not more true *esprit de corps* among doctors than among other classes—perhaps, indeed, there is less. A good many doctors, prompted by excellent intentions, have made efforts to unite their brethren together in a great attempt to improve their professional and general position. This is an eminently worthy object, and if carried out would work quite as much good to the public as to medical men. For it is a fact, which admits of no kind of denial, that half the medical men of Great Britain are grievously overworked and miserably underpaid. Now, an overworked and underpaid servant is by the very necessities of the case a bad servant. The public undoubtedly gets from overworked and underpaid medical men very inferior value for its money. Among the more recent of medical reformers is Dr. Rentoul, of Liverpool. Dr. Rentoul has worked hard and spent a good deal of money and time on the matter. Indeed, considering the decided want of gratitude shown to reformers generally, and the bad motives in addition attributed to medical reformers in particular, one cannot help wondering that he has continued to persevere in the work. At present we make no comment on the merits of his proposals. The medical men of South London have met, and have condemned them rather energetically, and perhaps precipitately. We shall take an early opportunity of dealing fully with the subject. In the meantime it seems desirable to bespeak for Dr. Rentoul the gratitude due to good motives and hard work in a worthy cause, and for his proposals a much more earnest and thorough consideration than they seem to have yet received. One of the debts which, according to Bacon, all medical men owe to their profession, is an earnest and determined attempt to lift one branch of it out of the despairing slough of overwork and underpay into which it has so deeply fallen.

**Children's Under-clothing.** — A morning contemporary a few days ago devoted a whole column to the subject of "Children's Dress." Every rational medical man will hail such an indication of public interest in so important a subject with great satisfaction. Not many years ago the children even of the rich were not thought to require underclothing, except of course one single thin garment of linen or cotton. No doubt many rheumatic fevers, many attacks of bronchitis, of pleurisy, and of pneumonia, many ruined lives, and many deaths resulted from the thoughtless and injurious custom. Of course mothers know better now, and the majority of them are not only willing to be instructed, but eagerly welcome every hint which will enable them to add to the comfort and safety of their girls and boys. Flannel or wool in some form, it is generally understood, should be next the skin, at any rate in autumn, winter, and spring. For our part we prefer it in summer too. But what form is the flannel or wool to take? It may take almost any form provided it covers both legs and arms, chest and throat, abdomen and back. But it ought not to be heavy, and it certainly should not be either hard or tight. Mothers, we think, will thank us for bringing to their notice a new kind of underclothing, which, while eminently suitable both for men and women, ought to be specially valuable for children. It is the new "woollen cellular" underclothing. Long ago cellular underclothing was made in cotton stuff, and suited many persons for summer wear very well. Now, however, and partly in consequence of a suggestion from the Medical Editor of this journal, the cellular clothing is made in fine soft woollen. The result is admirable. The clothing is extremely light, very elastic, and as warm and comfortable as possible. This is the very thing that is wanted for children. It is so light and elastic that it can be drawn round the throat so as to exclude the cold completely, without being in the least tight. Of its warmth and comfort we have satisfied ourselves by personal experiment. Mothers cannot do a better thing for their children in the cold season than to clothe them from head to foot with the "woollen," not cotton, "cellular" underclothing. It may be obtained, we believe, at the central depot, 75, Aldermanbury, E.C., or ordered through the family hosier.

